# A Systems-based Emergency Department Intervention to Conserve Intravenous Fluids During a National Shortage

**DOI:** 10.5811/westjem.53894

**Published:** 2026-06-16

**Authors:** Phillip D. Jenkins, Obert Xu, Kenneth DeVane, Melinda Hartenstein, Haley Manella, Beech Burns

**Affiliations:** Oregon Health & Science University, Department of Emergency Medicine, Portland, Oregan

## Abstract

**Background:**

Intravenous (IV) fluids are commonly used in emergency departments (ED), but their supply can be vulnerable to national shortages. In September 2024, Tropical Storm Helene disrupted production at a major manufacturing facility, triggering a nationwide IV fluid shortage. In response, our ED implemented a multiphase intervention aimed at reducing non-essential fluid utilization. We aimed to reduce total IV fluid use in the ED by at least 25% through a staged, systems-based conservation strategy.

**Methods:**

We conducted a single-center, retrospective, pre-post quality improvement initiative in a tertiary academic ED with approximately 57,000 annual visits. Interventions were deployed across three Plan–Do–Study–Act cycles: 1) clinician education on fluid stewardship (7 days); 2) protocol modifications to reduce routine IV placement (11 days); and 3) electronic health record (EHR) clinical decision support tools, including an interruptive alert and oral hydration order set (32 days). We analyzed daily fluid administration volumes, and ED census data from September 2024 (pre-intervention) and November 2024 (post-intervention). The primary outcome was total IV fluid usage per day; a secondary outcome was IV fluid volume per patient encounter. We compared mean daily utilization between periods using two-sample *t*-tests.

**Results:**

Average daily IV fluid use declined from 56.3 to 24.8 liters, a 56.0% relative reduction (*P* < .001), while ED volume remained stable (155.4 vs 153.1 encounters/day pre- vs post-intervention). Normalized per-encounter IV fluid administration decreased from 0.36 to 0.16 liters per visit (*P* < .001), representing a 56% relative reduction. Assuming a pharmacy acquisition cost of approximately $2 per liter of crystalloid, the intervention was associated with an estimated reduction of $63 in IV fluid expenditures per day (31.5 liters), or $1,890 over the 30-day post-intervention period.

**Conclusion:**

A structured intervention combining education, protocol changes, and EHR decision support significantly reduced IV fluid use in the ED without disrupting operations. This approach may serve as a scalable model for resource stewardship during future supply chain crises. The most substantial reductions occurred during the final intervention phase involving EHR-based decision support.

## INTRODUCTION

### Background

Intravenous (IV) fluids are a foundational therapeutic intervention in emergency medicine, frequently used for volume resuscitation, electrolyte management, and medication delivery.^1^ While often considered an abundant resource, the production and distribution of IV fluids are susceptible to supply chain disruptions.^2^,^3^ In September 2024, tropical storm Helene made landfall on the eastern seaboard of the United States, causing major flooding and structural damage to the Baxter manufacturing facility in North Carolina, the primary producer of IV fluid solutions for the U.S. market.^4^ This event precipitated a national shortage of IV fluids, prompting healthcare institutions across the country to implement emergency conservation strategies.

Historically, national fluid shortages have forced hospitals to ration use, delay elective procedures, or identify alternative therapies.^3^ However, many frontline clinicians are unaware of the volume of fluids administered daily or the potential for overuse in non-critical contexts. In emergency departments (ED), IV fluids are often initiated reflexively, even in low-acuity settings, despite evidence supporting the safety and efficacy of oral hydration in many cases.^5^,^6^ These patterns highlight an important opportunity for system-level interventions to improve resource stewardship without compromising patient care.

Recent literature has highlighted both the risks of IV fluid overuse and the operational consequences of national shortages. Stenson et al analyzed early lessons from the 2024 fluid shortage and emphasized the need for proactive inventory control, clinician education, and real-time analytics to guide conservation efforts.^7^ Similarly, Mahroos et al described systemic vulnerabilities in medication supply chains during disasters,^3^ while Aghaie et al found that physicians often underestimate fluid volumes delivered via IV antibiotics, further contributing to overuse.^6^ These studies underscore the importance of both awareness and structured stewardship interventions in the emergency setting.

### Local Problem

In the wake of the 2024 shortage, the ED at Oregon Health & Science University faced a declining stock of IV fluids and uncertain resupply timelines. Despite relatively stable patient volumes, the risk of exhausting available IV fluids posed a threat to safe and continuous emergency operations. An initial audit revealed widespread use of IV fluids for low-acuity complaints, extended maintenance infusions, and protocols that encouraged early IV access without clear indications. Leadership recognized an opportunity to address these practice patterns through targeted interventions.

### Objective

The goal of this quality improvement (QI) initiative was to reduce total IV fluid use in our ED by at least 25% during the national shortage. The team sought to implement a phased, systems-based conservation strategy that included educational messaging, protocol modification, and electronic health record (EHR) decision support. We hypothesized that such an approach could reduce overall IV fluid use without affecting ED volume or clinical operations, and that it could serve as a scalable model for future resource stewardship initiatives.

**Population Health Research Capsule**

What do we already know about this issue?

*Intravenous (IV) fluid supply is vulnerable to national shortages, and emergency departments (EDs) often overuse IV fluids in low-acuity patients despite safe oral alternatives*.

What was the research question?

*Can a staged ED intervention reduce IV fluid use during a national shortage?*

What was the major finding of the study?

*A staged ED intervention reduced IV fluid use 56% (56.3→24.8 liters/day; P < .001) with stable ED volume*.

How does this improve population health?

*Scalable ED stewardship can preserves scarce resources without harming operations during supply crises*.

## METHODS

### Study Design and Setting

This was a single-center, retrospective, pre–post QI initiative conducted in the ED at a tertiary academic medical center with an annual combined adult and pediatric ED volume of approximately 57,000 visits. The institutional review board deemed the project exempt from full board review as a QI activity.

### Intervention Strategy

**Figure 1:**
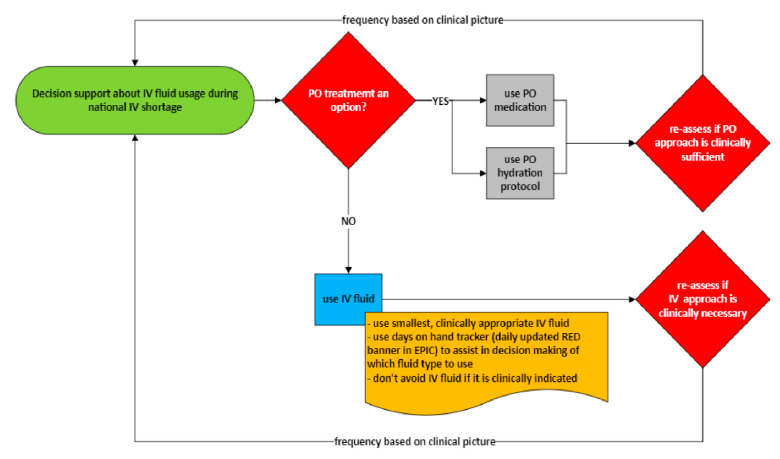
Decision-support flow diagram surrounding intravenous (IV) fluid use in a systems-based conservation intervention In the emergency. department. *IV*, intravenous; *PO*, per os

**Figure 2:**
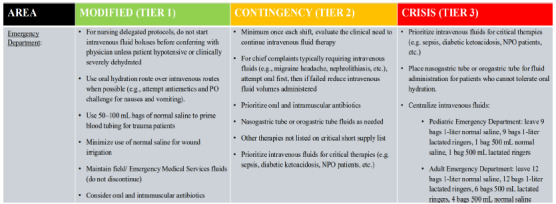
Tiered intravenous (IV) fluid conservation framework implemented in the emergency department during the national IV fluid shortage in 2024. The table outlines escalating stewardship strategies across three operational phases. Modified (Tier 1), Contingency (Tier 2), and Crisis (Tier 3), with progressively restrictive measures to preserve IV fluid supply. Interventions emphasize the preferential use of oral and intramuscular therapies, restriction of nonessential IV administration, and centralized fluid allocation during severe shortages. This framework guided clinical practice and resource distribution throughout the intervention period. *PO*, per os; *IV*, intravenous; *NPO*, nil per os.

We developed a staged fluid conservation approach informed by real-time supply levels, staff input, and institutional capacity. Interventions were designed and deployed using a Plan-Do-Study-Act (PDSA)^8^ framework. The timing and spacing of intervention phases were shaped by the operational urgency of an active national IV fluid shortage. To allow rapid deployment of education and protocol-level changes, PDSA cycles 1 and 2 were implemented in close succession, with limited opportunity for traditional wash-in or wash-out periods (Figures 1 and 2).

In contrast, PDSA Cycle 3 was deployed later due to the need for informatics development and testing and the institutional coordination required for EHR-based decision support tools. This variable pacing is consistent with the PDSA Model for Improvement, in which cycle duration reflects feasibility and infrastructure requirements for system-level change. Representative materials used during the PDSA cycles are provided in Appendix A–B.

### PDSA Cycle 1: Education and Best Practice Promotion

The first phase involved targeted education for clinicians and nurses on the principles of appropriate IV fluid stewardship. Messaging emphasized the use of enteral hydration for low-acuity patients, discouraged reflexive IV placement, and reviewed evidence-based thresholds for IV rehydration. These materials were shared during staff meetings, huddles, and email communication.

### PDSA Cycle 2: Protocol-based Conservation Measures

The second phase involved system-level changes, including modification of nurse-initiated protocols to limit automatic IV placement, encourage early IV-fluid discontinuation in stable patients, and promote oral or intramuscular antibiotic administration when clinically appropriate. Additionally, IV fluid stock was centralized at the institutional level, and volumes were distributed each day to match the estimated needs of individual units. Although additional fluids could be requested, this intervention was intended to mitigate overuse and discourage stockpiling.

### PDSA Cycle 3: Clinical Decision Support Tools

The final phase included deployment of an interruptive EHR-based alert encouraging oral rehydration for eligible patients and the implementation of a dedicated oral hydration order set. These tools were developed in collaboration with clinical informatics and were designed to promote conservation-compatible behaviors during order entry among clinicians.

### Data Collection and Outcome Measures

We obtained aggregate daily ED fluid-usage reports through the hospital’s analytics platform, which extracts administration volume data from the EHR. We collected patient census data from institutional dashboards, which were confirmed through internal reporting tools. The primary outcome was the average total volume of IV fluids administered per day across all ED patients. A secondary outcome was average fluid volume per patient per day, calculated by dividing total daily fluid volume by the number of ED encounters.

### Statistical Analysis

We compared pre-intervention data from September 2024 to post-intervention data from November 2024, reflecting one-month periods before and after full implementation of our QI initiative. Descriptive statistics were used to summarize fluid volume and ED census. We performed a two-sample *t*-test to compare mean daily fluid use between periods. A *P* value of < .05 was considered statistically significant. We prepared this manuscript in accordance with the Standards for Quality Improvement Reporting Excellence (SQUIRE 2.0) guidelines for reporting healthcare improvement studies. The intervention, context, study design, measures, analysis, results, and interpretation were structured to ensure transparency, reproducibility, and relevance for system-level improvement efforts. A completed SQUIRE checklist is available upon request.

## RESULTS

We evaluated IV fluid use in the ED surrounding the implementation of a fluid conservation protocol in response to national shortages. Pre-intervention data were collected September 1–30, 2024, and post-intervention data November 1–30, 2024, following full implementation of the staged conservation measures. The ED served a demographically diverse and stable population across all phases. Approximately half of the patients were female; 35% were 18–45 years of age; and 17–18% were ≥ 65 years of age. Racial and ethnic distributions of patients were consistent across periods, with approximately 76–78% White, 8–9% Black, 5–6% Asian, and 15–16% Hispanic, supporting generalizability of the findings.

### Primary Outcome: Reduction in Total Fluid Use

**Figure 3:**
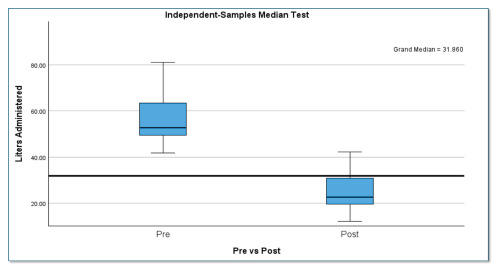
Distribution of total intravenous fluid volume administered per day before and after a systems-based conservation intervention in an academic emergency department during a national shortage.

The average daily IV fluid use decreased from 56.3 liters (L)/day in the pre-intervention period to 24.8 L/day following implementation of all interventions, representing a 56.0% absolute reduction from baseline (*P* < .001). This reduction occurred despite a stable average ED patient census of 155.4 encounters/day pre-intervention and 153.1 encounters/day post-intervention, confirming that the observed change was not attributable to fluctuations in patient volume (Figure 3).

### Per-Patient Fluid Use

Average daily IV fluid use declined from 56.3 to 24.8 L/day, a 56.0% absolute reduction (*P* < 0.001). The ED volume remained stable (155.4 vs 153.1 encounters/day pre- vs post-conservation intervention). Normalized per-encounter IV fluid administration decreased from 0.36 to 0.16 L/visit (*P* < .001), representing a 56% relative reduction.

### Weekly Trends and Stepwise Decline

**Figure 4:**
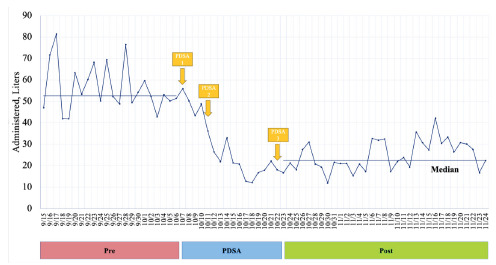
Weekly trends in intravenous (IV) fluid use during a systems-based conservation intervention in an academic emergency department. The line graph shows liters of IV fluid administered per week across the pre- and post-intervention periods, annotated with the timing of educational, protocol-based, and electronic health record decision support interventions. *PDSA*, Plan-Do-Study-Act.

A review of weekly use trends revealed a progressive decrease in fluid administration corresponding with the three phases of intervention. The earliest reductions were observed following department-wide education and best practice reminders (PDSA 1). A second stage (PDSA 2), which introduced conservation protocols triggered by stock levels, was followed by a more substantial decline. The most sustained reduction was observed after implementation of interruptive EHR-based clinical decision support tools and the addition of an oral hydration order set (PDSA 3) in late October 2024. This phased implementation was consistent with a cumulative and reinforcing effect of layered interventions. (Figure 4)

### Cost Implications

Based on an estimated pharmacy acquisition cost of approximately $2 per liter of crystalloid solution, the reduction of 31.5 L/day in average daily use corresponds to an approximate savings of $63 per day, or $1,890 across the 30-day post-intervention period. Implementation resources consisted primarily of clinician and operational staff time for educational efforts, protocol review, and EHR build, representing a one-time investment. Although we did not perform a formal cost-effectiveness analysis, these estimates suggest that the intervention was associated with a favorable balance between one-time implementation effort and ongoing reductions in IV fluid use.

### Operational Stability and Balancing Measures

Throughout the intervention period, ED operations and short-term safety indicators remained stable. Internal safety monitoring included a weekly review of return-visit rates, unplanned intensive care unit transfers, rapid-response activations, and mortality metrics via departmental quality dashboards and morbidity and mortality conferences. Mean daily ED volume was similar across phases (157.3 visits/day pre-intervention, 157.1 during PDSA cycles, and 153.6 post-intervention), and patient acuity distribution was unchanged, with Emergency Severity Index 1–2 encounters comprising approximately 30% of visits in all periods. Short-term outcomes also remained stable, including 48-hour return visit rates (3.84% pre- vs 3.98% post-intervention) and deaths within 24 hours of arrival (0.20% pre vs 0.16% post). These findings support the feasibility and acceptability of the conservation strategy without evidence of unintended negative consequences.

## DISCUSSION

In this single-center QI initiative, we implemented a phased fluid conservation strategy in the ED during a nationwide IV fluid shortage. Through education, protocol modification, and EHR-based decision support, we achieved a 56% absolute reduction in total IV fluid use without impacting patient volume. When normalized to ED census, fluid use per patient declined from 0.36 to 0.16 L/visit. These findings suggest that systems-based interventions can meaningfully reduce resource use during supply chain disruptions, even in high-acuity settings such as the ED.

### Interpretation of Findings

The success of this initiative likely reflects the synergistic impact of multiple interventions. Early education helped to raise awareness and shift the culture regarding reflexive fluid use. Modifications to nurse-initiated protocols and centralization of supply stock created structural friction that discouraged low-value use. The most pronounced reduction followed deployment of interruptive EHR alerts and the addition of an oral hydration order set, reinforcing the existing literature demonstrating that clinical decision support tools have a particularly strong influence on clinician behavior.^9^,^10^ This layered approach aligns with other studies showing that combining education with workflow and system-level changes is more effective than isolated interventions.^10^–^13^

Intervention timing reflected operational feasibility: education and protocol changes were implemented rapidly during the shortage, whereas EHR-based tools required longer informatics development and approval timelines. This sequencing likely contributed to the stepwise reduction observed and highlights a practical consideration for teams aiming to generalize or replicate similar conservation efforts. Our findings also highlight how clinical practice norms, such as routine IV placement or prolonged administration of maintenance fluids, can persist despite evidence of limited benefit. By disrupting these default patterns, we encouraged a more intentional approach to hydration and medication administration. Importantly, the interventions targeted process rather than clinician judgment: clinicians retained autonomy but were prompted to reconsider standard practices considering the shortage.

### Implications for Practice

This initiative demonstrates that significant reductions in fluid use are feasible, even in busy, complex clinical environments. Our approach may serve as a blueprint for other institutions facing similar shortages or seeking to promote sustainable resource use. Although this project was initiated in response to an acute supply chain disruption, the strategies deployed, especially EHR-based prompts and protocol review, may have ongoing value in promoting high-value care beyond the context of crisis. Furthermore, this work highlights the potential for EDs to serve as testbeds for rapid-cycle quality improvement and systems redesign.

## LIMITATIONS

This study has several limitations. First, it was conducted at a single academic institution and may not generalize to community EDs or non-academic settings. Second, we evaluated only short-term outcomes and could not assess the durability of fluid conservation over time. We did not differentiate among crystalloid types administered (eg, lactated Ringer’s vs normal saline), which may have important cost and usage implications, particularly given the higher acquisition cost and more limited availability of balanced solutions. Finally, our pre-post study design limited our ability to draw definitive causal inferences. Future studies could evaluate specific patient-safety centered balancing measures to assess for unintended consequences.

## CONCLUSION

This quality improvement initiative demonstrated that a structured, systems-based approach to IV fluid conservation can lead to significant reductions in use during times of critical supply shortage. As supply chain disruptions become increasingly common, scalable and sustainable models like ours may serve as valuable frameworks for institutions seeking to promote high-value care under constrained conditions.

## Supplementary Information
