# Supplementary material for: A Systems-based Emergency Department Intervention to Conserve Intravenous Fluids During a National Shortage
Source: West J Emerg Med. 2026 Jun 16;27(4):855–60. doi: 10.5811/westjem.53894 (PMC13436644; doi:10.5811/westjem.53894)
Supplement: Supplementary file 1 [file wjem-27-855-s001.pdf]

Decision Support about IV Fluid

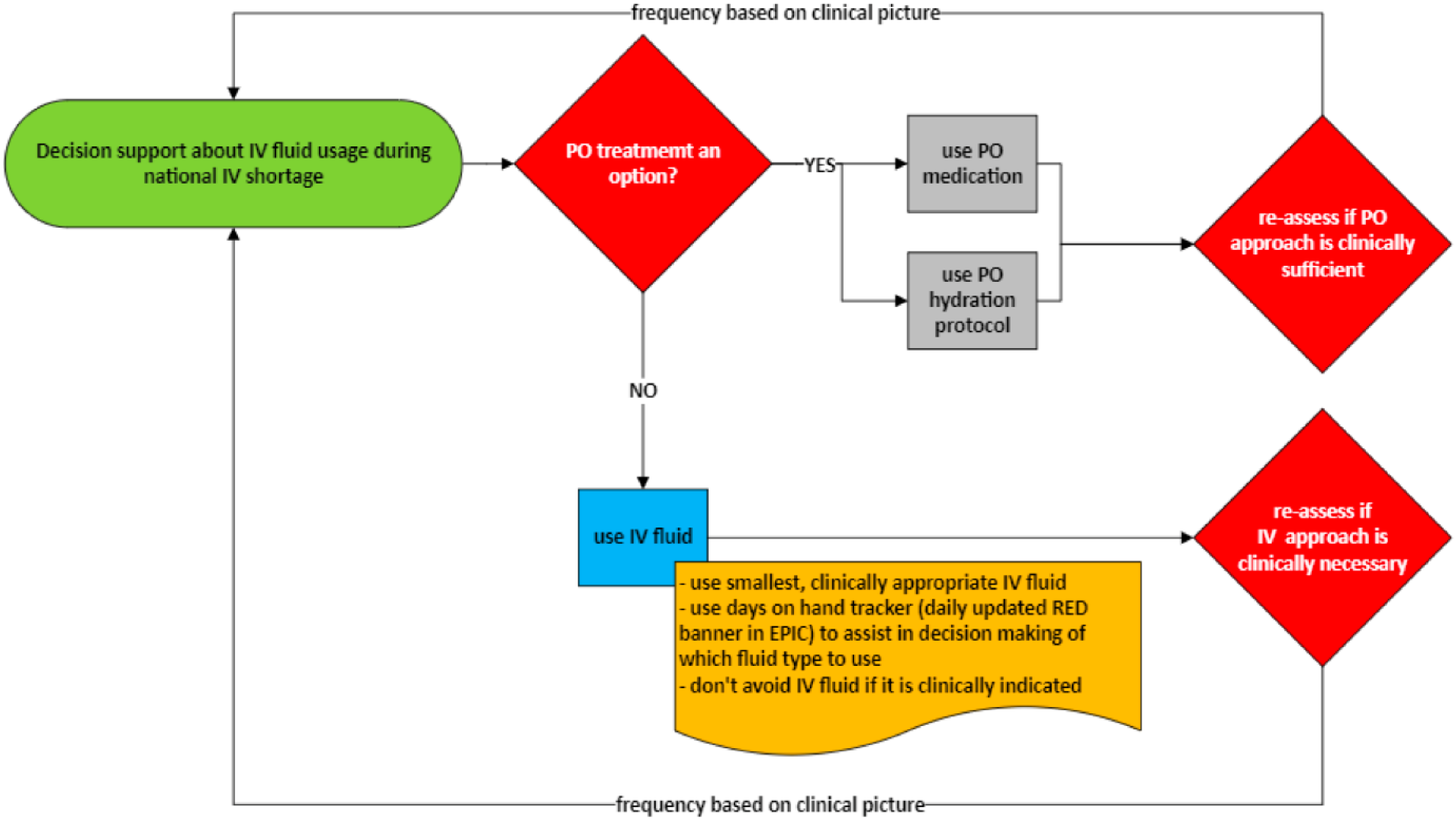

Source: Oregon Health and Science University
